# Physical activity and sedentary behavior among Chinese children aged 6–17 years: a cross-sectional analysis of 2010–2012 China National Nutrition and health survey

**DOI:** 10.1186/s12889-019-7259-2

**Published:** 2019-07-11

**Authors:** Chao Song, Weiyan Gong, Caicui Ding, Fan Yuan, Yan Zhang, Ganyu Feng, Zheng Chen, Ailing Liu

**Affiliations:** Department of Nutrition and Health Education, National Institute for Nutrition and Health, Chinese Center for Disease Control and Prevention, No. 27 Nanwei Road, Xicheng District, Beijing, 100050 China

**Keywords:** China, Physical activity, Sedentary behavior, Children

## Abstract

**Background:**

Patterns of physical activity (PA) and sedentary behavior have important implications for child health. The purpose of the study was to describe the PA and sedentary behavior among Chinese children.

**Methods:**

The study analyzed the PA and sedentary behavior status of 38,744 Chinese children aged 6–17 years, based on the data from China National Nutrition and Health Survey (CNNHS) in 2010–2012.

**Results:**

Chinese children spent 61.6 min/d on school-time PA and the proportion with 60 min and over on school-time PA was 52.5%. The average transportation time was 38.5 min/d and the main mode for children was walking (50.9%), followed by taking private vehicles (19.4%), taking public transportation (16.4%) and cycling (13.3%). Approximately 70% Chinese children did domestic PA and they spent 17.2 min/d on it. Children spent 2.9 h per day on leisure-time sedentary behaviors and 85.8% of them engaged in sedentary behaviors longer than 2 h/d. The proportion of children participating in leisure-time PA was 35.4%and they performed moderate-to-vigorous PA on 3.4 days per week with average 44.9 min per day. Boys were more active in school-time PA, leisure-time PA and transportation, while girls spent more time on domestic PA and homework. More older children took active transportation and spent more time on domestic PA, leisure-time PA and sedentary behaviors compared with younger children. Children in urban area were more likely to take inactive transportation and participate in leisure-time PA, spent less time on domestic PA while more time on sedentary behaviors compared with their counterpart.

**Conclusions:**

Chinese children performed little physical activity and spent long time engaging in sedentary behaviors. Their physical activity and sedentary behaviors varied by gender, age and area.

## Background

Globally, the number of obese children and adolescents (aged five to 19 years) has risen tenfold in the past four decades [1]. In 2016, there were 50 million girls and 74 million boys with obesity in the world [2]. Owing to the well-established childhood obesity epidemic, the population prevalence of high blood pressure in the young is increasing [3]. Recently reports revealed the emerging problem of type 2 diabetes in children and adolescents [4, 5]. Accumulated evidence suggested that physical activity (PA) and sedentary behaviors were associated with physical fitness and mental health in childhood [6–9]. The World Health Organization (WHO) defined PA as any bodily movement produced by skeletal muscles that required energy expenditure, including activities undertaken while working, playing, carrying out household chores, travelling, and engaging in recreational pursuits [10]. However, a large percentage of children did not perform recommended levels of PA. Globally, 81% of the world’s adolescents aged 11–17 years were insufficiently physically active in 2010 [10].

Independent of PA levels, sedentary behavior is associated with increased risk of obesity, and has a negative impact on several health outcomes [6, 11–15]. However, an ever-increasing sedentary lifestyle is becoming widespread among children [11].

China has experienced a rapid social and health transition nowadays, which made a significant impact on health and lifestyle among Chinese children, and some health related problems emerged. In Chinese school-aged children, only 3 in 10 children achieved an “excellent” or “good” fitness standard in 2016 [16]. Meanwhile, the overweight and obesity prevalence of Chinese children aged 6–17 years were 9.6 and 6.4% respectively according to the results of China National Nutrition and Health Survey(CNNHS), which presented an increase compared with the result of China National Nutrition Survey in 2002 [17, 18]. The trend also has been reported in other Chinese studies [19, 20]. Additionally, some non-communicable disease arose among Chinese children, for instance the prevalence of hypertension was 12.4% in Chinese children aged 6–17 years [21]. The pattern of PA and sedentary behavior was one of most fundamental risk factors. The current study was based on the data from CNNHS in 2010–2012, the largest and most comprehensive study of nutrition and health outcomes ever conducted in China. The purpose of the study was to describe the status of PA and sedentary behavior of Chinese children, investigate how PA and sedentary behavior varied by gender, age and area, then to provide basic information to aid in developing PA intervention strategies.

## Methods

### Sampling method and implementation

The CNNHS was a nationally representative cross-sectional study conducted by the National Institute for Nutrition and Health, Chinese Center for Disease Control and Prevention (NINH, China CDC) to assess the health and nutrition status of Chinese residents. The 2010–2012 survey covered all 31 provinces, autonomous regions, and municipalities directly under the central government throughout China (except for Taiwan, Hong Kong, and Macao). The estimated sample size was 36000 children aged 6–17 years and a multi-stage stratified cluster random sampling method was used in CNNHS. A total of 150 sites were selected, among which 34 were big cities, 41 were medium and small cities, 45 were ordinary rural areas, and 30 were poor rural areas. The four areas were classified according to the economy and social development. Big cities were the central urban areas of big cities including municipalities, 6 designated cities and capital cities (population more than one million). Medium and small cities were downtown areas that excluded the big cities. Poor rural areas were the key poor counties identified by China. Ordinary rural areas were all the rest counties outside the poor rural areas. In each site, six villages or communities were randomly selected, and in each village or community, 75 households were randomly selected.

The children aged 6–17 years in each family were involved in this survey. If the number of children in each age group was less than 20 (the number of boys and girls was equal), some children would be selected from nearby primary schools to meet the minimum sample size. Finally a total of 38,744 children aged 6–17 years was involved and all of them completed the survey. The protocol of the 2010–2012 CNNHS was approved by the Ethical Committee of NINH, China CDC (2013–018). Signed consent forms were obtained from the children’s parents or legal guardians before data collection.

### Physical activity and sedentary behaviors assessment

An interview-administered questionnaire which conducted at child’s home or school by trained investigators was used to collect the PA and sedentary behaviors in the past semester. For the children younger than 10 years old, questionnaires were completed with the assistance of their parents or their main caregivers. The study assessed four PA domains: school-time PA, transportation PA, leisure-time PA and domestic PA. School-time PA included physical education class, exercise during class breaks, etc. Transportation PA included walking, cycling, taking public transport (bus, subway and school bus), taking private vehicles (cars, taxi, motorcycle, electric vehicles or bicycles). Leisure-time PA included planned, structured and repetitive moderate-to-vigorous PA (MVPA) in the leisure time out of school, the purpose of which was to maintain and improve fitness and health. The total time of MVPA (exceeding 10 min each time) and vigorous PA (VPA, exceeding 20 min each time) was collected separately. Domestic PA was defined as any housework that involves physical activity, such as cleaning the house, cooking, doing laundry, and so on.

Leisure-time sedentary behaviors included watching television, using computers, playing video games, reading and doing homework in leisure time. The total time on all sedentary behaviors was collected.

### Statistical analysis

The participants were divided into sub-groups according to their gender, age group and areas. Age and gender standardization were performed based on the population of China in 2009 published by National Statistics Bureau. The subgroup differences in school-time PA, transportation PA, domestic PA, leisure-time sedentary behaviors were assessed using the survey-weighted regression analysis. The subgroup difference in the proportions and their 95% confidence Intervals (95% CIs) were compared using the Rao-Scott Chi-square test. For leisure-time PA (MVPA and VPA), t tests were used to compare means between different gender and age groups. Group comparisons among different areas were performed using variance analysis. The significance level was set at *p* < 0.05 using two-sided tests.

The statistical software package SAS version 9.4 (SAS Institute Inc., Cary, NC, USA) was used for data analysis.

## Results

### Characteristics of participants

Table 1 presents the demographic characteristics of the participants studied. The sample size was 38744 (boys: 19631, girls: 19113) with the mean age being 11.8 ± 3.3 years.

**Table 1:** Basic information of participants

	N(%)
Total	38744
Gender	
Boys	19631(50.7)
Girls	19113(49.3)
Age(years)	
6–12	23456(60.5)
13–17	15288(39.5)
Areas	
Big Cities	8646(22.3)
Medium and Small Cities	10825(27.9)
Ordinary rural areas	12130(31.3)
Poor rural areas	7143(18.5)

### School-time activities

Chinese children spent 5.2 days in school per week. Older children spent more time in school (5.3 day vs. 5.0 day, *P* < 0.0001) compared to younger ones. The average school-time PA was 61.6 min per school day with higher in boys than in girls (62.3 min vs. 60.8 min, *P* = 0.0093). The proportion of children who spent more than 60 min on school-time PA was 52.5%, and there was no significant difference between different gender, age groups and areas (Table 2).

**Table 2:** Physical activity and sedentary behavior of Chinese children aged 6–17 years

	Total	Gender		P	Age(years)		P	Areas				P
		Boys	Girls		6–12	13–17		Big Cities	Medium and small Cities	Ordinary rural areas	Poor rural areas	
School days per week^a^	5.2 ± 0.02	5.2 ± 0.02	5.2 ± 0.02	0.7263	5.0 ± 0.01	5.3 ± 0.03	< 0.0001	5.1 ± 0.03	5.1 ± 0.02	5.2 ± 0.03	5.2 ± 0.047	0.0531
school-time PA(min/d)^a^	61.6 ± 1.71	62.3 ± 1.87	60.8 ± 1.57	0.0093	60.6 ± 1.38	62.8 ± 2.36	0.1585	60.2 ± 2.61	64.8 ± 3.43	58.0 ± 2.11	61.6 ± 3.098	0.3848
school-time PA ≥ 60 min/d(%,95*CI*)	52.5 (48.3~56.7)	52.8 (48.6~57.1)	52.1 (47.9~56.3)	0.2801	52.3 (48.0~56.6)	52.7 (48.1~57.4)	0.7932	49.9 (43.3~56.4)	56.0 (49.6~62.5)	51.4 (43.9~58.9)	47.0 (37.0~56.9)	0.3412
Transportation time (min/d)^a^	38.5 ± 0.83	38.6 ± 0.84	38.4 ± 0.87	0.5563	37.7 ± 0.90	39.5 ± 1.01	0.0494	41.9 ± 1.15	38.7 ± 1.34	35.4 ± 1.23	43.3 ± 2.549	0.0007
Transportation form(%,95*CI*)				0.0003			< 0.0001					< 0.0001
Walking	50.9 (47.3~54.5)	51.0 (47.3~54.6)	50.8 (47.2~54.4)		55.6 (51.6~59.7)	45.0 (41.3~48.8)		46.8 (41.4~52.2)	45.2 (39.6~50.7)	48.7 (42.1~55.4)	70.6 (62.7~78.6)	
Cycling	13.3 (11.2~15.4)	14.1 (12.0~16.2)	12.3 (10.2~14.5)		7.4 (5.9~8.8)	20.5 (17.2~23.8)		11.3 (7.8~14.88)	14.6 (11.0~18.3)	14.5 (10.8~18.2)	8.2 (4.9~11.6)	
Public transit	16.4 (14.1~18.7)	16.1 (13.8~18.5)	16.7 (14.3~19.1)		11.4 (9.2~13.6)	22.6 (19.1~26.0)		24.6 (21.3~27.8)	17.1 (13.1~21.1)	17.0 (13.2~20.8)	10.6 (6.0~15.1)	
Private vehicles	19.4 (16.7~22.1)	18.8 (16.1~21.5)	20.1 (17.3~22.9)		25.6 (22.0~29.2)	11.9 (9.6~14.1)		17.3 (13.5~21.1)	23.1 (18.1~28.1)	19.8 (15.8~23.8)	10.5 (5.0~16.1)	
Domestic PA(%,95*CI*)	69.6 (66.8~72.4)	66.1 (63.0~69.1)	73.6 (71.0~76.3)	< 0.0001	62.5 (59.0~65.9)	78.4 (75.9~80.9)	< 0.0001	73.3 (70.0~76.5)	68.9 (64.6~73.1)	67.9 (62.3~73.5)	73.6 (68.2~79.1)	0.3238
Domestic PA time(min/d)^a^	17.2 ± 0.66	15.7 ± 0.70	18.9 ± 0.66	< 0.0001	13.6 ± 0.58	21.6 ± 0.85	< 0.0001	17.8 ± 1.09	15.5 ± 0.81	16.1 ± 0.96	23.3 ± 2.685	0.0274
Sedentary time(h/d)^a^	2.9 ± 0.04	2.9 ± 0.04	2.9 ± 0.05	0.7832	2.6 ± 0.04	3.2 ± 0.07	< 0.0001	3.2 ± 0.05	3.0 ± 0.07	2.8 ± 0.07	2.7 ± 0.111	< 0.0001
Sedentary time ≥ 2 h/d(%,95*CI*)	85.8 (83.8~87.7)	85.9 (83.9~87.9)	85.6 (83.6~87.6)	0.3755	83.0 (80.7~85.2)	89.2 (87.1~91.2)	< 0.0001	90.0 (88.0~91.9)	86.6 (83.5~89.7)	86.4 (83.3~89.5)	80.9 (75.3~86.5)	0.0505
Homework time(h/d)^a^	1.5 ± 0.03	1.4 ± 0.03	1.5 ± 0.03	< 0.0001	1.2 ± 0.02	1.8 ± 0.05	< 0.0001	1.8 ± 0.05	1.6 ± 0.06	1.4 ± 0.05	1.3 ± 0.062	< 0.0001

^a^Data was described as mean ± SE

### Transportation PA

The average transportation time of Chinese children was 38.5 min/d, which was higher in older children (*P* = 0.0494), and children from different areas spent different time on transportation (*P* = 0.0007) (Table 2).

Children commuted to school mainly by walking (50.9%), followed by taking private vehicles (19.4%), taking public transit (16.4%) and cycling (13.3%). The transportation mode varied by gender, age and area as shown in Table 2.

### Domestic PA

The proportion of Chinese children doing domestic PA was 69.6%, which was higher in girls (73.6% vs. 66.1%, *P* < 0.0001), older children (78.4% vs. 62.5%, *P* < 0.0001). The average time on domestic PA was 17.2 min/d, which was higher in girls (18.9 vs. 15.7 min/d, *P* < 0.0001), older (21.6 vs. 13.6 min/d, *P* < 0.0001) and poor rural area (*P* = 0.0274) (Table 2).

### Leisure-time sedentary behavior

The average time children spent on leisure-time sedentary behavior was 2.9 h/d and the proportion of children engaging in leisure-time sedentary behavior longer than 2 h/d was 85.8% (Table 2). The older children and the children in big cities spent more time on leisure-time sedentary behavior compared with their counterparts, while no significant difference was found between boys and girls (Table 2). Children spent nearly 1.5 h/d on their homework which accounted for about a half of the sedentary time. Girls, older children and children in big cities spent more time on homework compared with their counterparts (Table 2).

### Leisure-time PA

Table 3 shows that 35.4% of Chinese children participated in leisure-time PA. The proportion of leisure-time PA participation was higher in boys and older age group (*P* < 0.0001). The highest proportion of leisure-time PA was found in children from big cities, and the lowest proportion in children from poor rural areas (*P* < 0.0001).

**Table 3:** Physical activity of Chinese children aged 6–17 years in their leisure time

	Total	Gender		P	Age(years)		P	Areas				P
		Boys	Girls		6–12	13–17		Big Cities	Medium and Small Cities	Ordinary rural areas	Poor rural areas	
Leisure-time PA participation(%,95*CI*)	35.4 (32.1~38.7)	37.8 (34.4~41.3)	32.6 (29.3~35.9)	< 0.0001	31.7 (28.3~35.1)	39.9 (36.0~43.8)	< 0.0001	54.0 (50.2~57.8)	39.4 (33.9~44.9)	33.0 (27.6~38.5)	23.5 (15.5~31.5)	< 0.0001
MVPA(day)^a^	3.4 ± 1.87	3.5 ± 1.90	3.3 ± 1.84	< 0.0001	3.4 ± 1.85	3.4 ± 1.90	0.7778	3.3 ± 1.96	3.3 ± 1.90	3.4 ± 1.79	3.5 ± 1.93	< 0.0001
t/F value		5.23			0.28			18.49				
MVPA (min/day)^a^	44.9 ± 36.35	47.2 ± 38. 43	42.3 ± 33.60	< 0.0001	43.9 ± 34.28	46.2 ± 38.80	0.0002	48.2 ± 39.66	44.9 ± 38.34	37.6 ± 28.43	46.5 ± 37.32	< 0.0001
t/F value		8.15			−3.78			11.78				
VPA(day)^a^	2.3 ± 1.57	2.4 ± 1.59	2.3 ± 1.55	0.002	2.3 ± 1.48	2.4 ± 1.67	< 0.0001	1.6 ± 1.71	1. 6 ± 1.73	1.4 ± 1.53	2.1 ± 1.79	< 0.0001
t/F value		3.10			−5.00			16.72				
*P*												

^a^Data was described as mean ± SD

The children performed MVPA on 3.4 days per week with average 44.9 min per day, and performed VPA on 2.3 days per week. The leisure-time PA varied by gender, age groups and areas (*P* < 0.0001), except for the amount of day on MVPA between different age groups (*P* = 0.7778).

## Discussion

This study clearly demonstrated different domains of PA of Chinese children. As regards of school-time PA, WHO recommended children and youth aged 5–17 should accumulate at least 60 min of MVPA daily [22]. This study showed that only 52.5% of children spent at least an hour on physical activity in school. This results suggested approximately half children did not meet the recommendation of WHO, and boys spent more time doing School-time PA in comparison with girls, so Chinese children, especially girls, should be encouraged to participate PA in their leisure time.

Our data showed that Chinese children spent 38.5 min on their commuting, which was similar to the result of national nutritional survey in 2002 [18]. The results also showed 64.2% children walked or cycled to school, which was higher than US children and lower than that in Swiss [23, 24]. With urbanization and economic development, a declining trend of active transport (walking and cycling) was observed internationally [23–25]. This study also showed that in comparison with children in rural areas, fewer children in urban areas went to school by active transport, while more of them went to school by motorized vehicles. Similar trends have been found in United States, New Zealand and African children [26–28]. Commuting is one type of PA. It has been widely acknowledged that walking and cycling to school can contribute to accumulate PA [29–32], so these active means of school transport should be encouraged.

According to the current study, only 35.4% of Chinese children performed leisure-time PA. The data indicated that Chinese children were physically inactive in their leisure time, and the similar problem existed in many other countries: as described by Hallal e tal 2012, and has been found among students aged 13–15 in the Association of Southeast Asian Nations (ASEAN) member states, in Portuguese healthy children and adolescents aged 10–18 years, and in Scottish adolescents [33–36]. Boys participated in more PA, and which was similar in several previous studies. For example, a study conducted on a nationally representative sample of US students in grades 6–10 showed that boys reported significantly more PA than girls. A cross-sectional study including 1568 UK children aged 9–10 years showed that girls participated less in VPA and had more sedentary time than boys. Another study conducted in Tehran high schools showed that boys had significantly higher daily energy consumption than girls [37–39].

For children and adolescents, doing domestic PA can foster their problem-solving abilities and help them develop self-confidence and a sense of responsibility, so children and adolescents should undertake some housework. In the current study, approximately 70 % Chinese children did domestic PA, which was higher than that of the survey in 2002 [18]. Data showed Chinese children aged 6–12 years spent 13.6 min/d on domestic PA, which is a little higher than that in 2002 [18]. Chinese children aged 13–17 years spent 21.6 min/d, which is lower than that in 2002 and lower than American children aged 15–17 years (0.78 h) [18, 40].

This study showed that Chinese children spent 2.9 h on leisure sedentary behaviors. Actually, in children’s daily lives, there was quite a lot of time spent on sitting in classes, which was basically sedentary. If these periods were added, sedentary time would be longer. There was no significant difference between boys and girls, which was similar to the results in 2002 [18], Canadian children [41], and UK children aged 9–11 years [8]. Children in higher age groups spent more time on their sedentary behaviors and homework than the lower age groups, and this difference was also found in a New Zealand-based study and Pate’s study [42, 43], which was likely due to the greater learning pressure on the senior children to learn and to achieve high grades. Children in urban areas had more leisure sedentary time than that in rural areas. In contrast, children in urban areas had a greater chance to access electronic equipment such as television, computers, smart phones and others. Similarly, a survey in Brazil found that the children in urban areas spent more time on television, video games and computers [44]. The Sport, Physical Activity, and Eating Behavior: Environmental Determinants in Young People study in the UK showed that children from higher socio-economic status families exhibited more after-school and weekend sedentary time [45]. In addition, similar results, that affluence was a significant contributing factor to sedentary behavior, were found in Ghanaian adolescents [46]. Moreover, children in urban areas spent more time on homework than that in rural areas. This may be because urban parents paid more attention to the children’s education, and their children had more homework to do. Therefore, supportive family environments need to be established to reduce sedentary time.

The data gave a general description of PA and sedentary behavior of Chinese children aged 6–17 years, and provided a basis and a reference for further intervention strategies. However, the data were collected through self- or proxy-reports, so our study suffered from reporting bias, specifically a combination of social desirability bias and the cognitive challenge associated with estimating physical activity for adults and children [47]. However, before the survey, a pilot study was performed to ensure the survey was scientific and feasible.

In China, the government have made some regulations and taken some actions. For example, School Sanitary Regulation issued by Ministry of Education and National Health Commission in 1990 and Sun Sports Programme launched in 2007. The current study indicated the level of PA among children and adolescents was still far from optimistic, especially leisure-time PA, as well as higher time of sedentary behaviors. The reason might due to the current policy mainly aimed at school-time PA, rather than the each domain of PA and sedentary behaviors. The transportation PA, leisure-time PA and domestic PA are also important components of PA and good to the development and health of children, and on the contrast, sedentary behavior is an independent risk factor. So the attention should be also paid outside the school, and the supportive environments such as the community and the family should be established.

## Conclusions

Our data suggested that only one in three children participated leisure-time PA, two in three children went to school by active transport, and about 70 % children did domestic PA. Chinese children spent long time on sedentary behavior. Boys were more active to participate in PA than girls, while girls spent more time on domestic PA and homework. Older children and children in rural area were more likely to take active transportation compared with their counterparts, and older children and children in urban areas spent more time on sedentary behaviors. So a supportive environment should be established at different levels such as government, community, schools and families, to encourage children to promote their physical activity levels and reduce their sedentary behavior, especially for girls, older children and children in urban area.

## Data Availability

The datasets generated and/or analyzed during the current study are not publicly available because some results are still being analyzed but are available from the corresponding author on reasonable request.

## Abbreviations

95% CIs: Confidence Intervals
ASEAN: The Association of Southeast Asian Nations
CNNHS: China National Nutrition and Health Survey
MVPA: Moderate-to-vigorous PA
NINH, China CDC: National Institute for Nutrition and Health, Chinese Center for Disease Control and Prevention
PA: Physical activity
VPA: Vigorous PA
WHO: World Health Organization
